# The bacterial community structure and functional profile in the heavy metal contaminated paddy soils, surrounding a nonferrous smelter in South Korea

**DOI:** 10.1002/ece3.4170

**Published:** 2018-05-20

**Authors:** Sherlyn C. Tipayno, Jaak Truu, Sandipan Samaddar, Marika Truu, Jens‐Konrad Preem, Kristjan Oopkaup, Mikk Espenberg, Poulami Chatterjee, Yeongyeong Kang, Kiyoon Kim, Tongmin Sa

**Affiliations:** ^1^ Department of Environmental and Biological Chemistry Chungbuk National University Cheongju Korea; ^2^ Institute of Ecology and Earth Sciences University of Tartu Tartu Estonia; ^3^ Present address: Department of Biology Benguet State University La Trinidad Philippines

**Keywords:** bacterial community, functional profiles, heavy metal contamination, pyro‐sequencing

## Abstract

The pollution of agricultural soils by the heavy metals affects the productivity of the land and has an impact on the quality of the surrounding ecosystems. This study investigated the bacterial community structure in the heavy metal contaminated sites along a smelter and a distantly located paddy field to elucidate the factors that are related to the alterations of the bacterial communities under the conditions of heavy metal pollution. Among the study sites, the bacterial communities in the soil did not show any significant differences in their richness and diversity. The soil bacterial communities at the three study sites were distinct from one another at each site, possessing a distinct set of bacterial phylotypes. Among the study sites, significant changes were observed in the abundances of the bacterial phyla and genera. The variations in the bacterial community structure were mostly related to the general soil properties at the phylum level, while at the finer taxonomic levels, the concentrations of arsenic (As) and lead (Pb) were the significant factors, affecting the community structure. The relative abundances of the genera *Desulfatibacillum* and *Desulfovirga* were negatively correlated to the concentrations of As, Pb, and cadmium (Cd) in the soil, while the genus *Bacillus* was positively correlated to the concentrations of As and Cd. According to the results of the prediction of bacterial community functions, the soil bacterial communities of the heavy metal polluted sites were characterized by the more abundant enzymes involved in DNA replication and repair, translation, transcription, and the nucleotide metabolism pathways, while the amino acid and lipid metabolism, as well as the biodegradation potential of xenobiotics, were reduced. Our results showed that the adaptation of the bacterial communities to the heavy metal contamination was predominantly attributed to the replacement process, while the changes in community richness were linked to the variations in the soil pH values.

## 1 INTRODUCTION

Although heavy metals are naturally present in the soil, geological, and anthropogenic activities increase the concentrations of these elements to amounts that are harmful to both plants and animals. Some of these activities include the mining and the smelting of metals, the burning of fossil fuels, the use of fertilizers and pesticides in agriculture, the production of batteries, and other metal products in industries, sewage sludge, and municipal waste disposal (Chibuike, Obiora, Chibuike, & Obiora, 2014). The soils act as major sinks for the heavy metals that constitute an ill‐defined group of inorganic chemical hazards. The most commonly found heavy metals at the contaminated sites are lead (Pb), chromium (Cr), arsenic (As), zinc (Zn), cadmium (Cd), copper (Cu), mercury (Hg), and nickel (Ni) (Wuana & Okieimen, 2011). The heavy metals do not undergo microbial or chemical degradation, persisting for a long time after their introduction into the soil (Adriano, 2001). The pollution of the soils by the heavy metals poses a serious threat to ecosystems and human health (Liu et al., 2016). Studies have shown that the pollution by heavy metals affects plant growth and genetic variation, changes the composition of the soil microbial community, and reduces microbial activity (Ellis, Morgan, Weightman, & Fry, 2003; Hong, Si, Xing, & Li, 2015; Li et al., 2006; Linton, Shotbolt, & Thomas, 2007; Moffett et al., 2003; Müller, Westergaard, Christensen, & Sørensen, 2001; Singh et al., 2014; Xie et al., 2016; Zhou et al., 2011). The resilience and metal tolerance mechanisms, allowing the microbial populations not only to survive but also maintain the main functions of their communities, have been observed in the soil that has a long‐term history of heavy metal contamination (Azarbad et al., 2015, 2016). Metabolically, the plastic bacterial groups are relatively more abundant in the Pb‐Zn contaminated mine soils and several functions, including those related to the metal resistance mechanisms, are highly expressed (Epelde, Lanzén, Blanco, Urich, & Garbisu, 2015). The changes in the bacterial communities are shown to be complex in the polluted soils. For example, the phosphorus solubilization capability of the microbial communities is related to the mobility of the heavy metals (Pb and Cd) in a long‐term contaminated soil (Yuan et al., 2017). The soil microbial community structure and activity are known to be determined to a great extent by the soil type as well as the ecosystem type (Girvan, Bullimore, Pretty, Osborn, & Ball, 2003; Schneider et al., 2017; Truu, Truu, & Ivask, 2008). Therefore, the different responses by the microbial communities to heavy metal pollution can be expected in the different soils with varying management histories (Azarbad et al., 2016). Several studies have described the quantitative and/or qualitative changes in the bacterial communities, in response to the increase in the concentrations of the different heavy metals in the agricultural soils and forest soils (Åkerblom, Bååth, Bringmark, & Bringmark, 2007; Cruz‐Paredes, Wallander, Kjøller, & Rousk, 2017; Igalavithana et al., 2017; Pennanen, Frostegard, Fritze, & Baath, 1996; Sharaff, Kamat, & Archana, 2017). Some of these studies have also correlated the structural changes to the soil functions such as enzymatic activities (Åkerblom et al., 2007; Borowik, Wyszkowska, & Wyszkowski, 2017). Even microbial enzymes have been proposed to be used as the early warning tools for monitoring the soils at mining sites (Wahsha et al., 2017). Recent studies have revealed that some bacterial groups such as *Verrucomicrobia* are important in carbon cycling, respond to the high content of Pb in the soils of the different forest ecosystems, and can be used as the indicators of heavy metal pollution in the soils (*Verrucomicrobia/Chlamydiae* ratio; Schneider et al., 2017).

Already, the elevated levels of the heavy metals in the agricultural soils have been a worldwide concern for several decades (Ashraf et al., 2017; Igalavithana et al., 2017; Ippolito, Ducey, & Tarkalson, 2010; Nriagu, 1998). Rice is a staple food in most of the Asian countries with 70% of the total food‐derived energy coming from rice (Phuong, Chuong, & áTong Khiem, 1999). However, the paddy fields are prone to the exposure to heavy metals and the repeated consumption of rice from the paddy fields, contaminated by the heavy metals, leads to serious health disorders (Kunito et al., 2017; Xiao et al., 2017; Xu et al., 2017). In this context, the knowledge about the functioning of these ecosystems is most important. Currently, the microbial communities of paddy soils are intensively studied using most advanced molecular techniques but mostly with respect to the different agricultural management options. So far, the microbiological research carried out in the paddy soils with regard to heavy metal pollution is scarce and are mostly based on low‐resolution methods (denaturing gradient gel electrophoresis [DGGE]) for microbial community assessment and gives controversial results. In one short‐term study, a decrease in the diversity of the bacterial community and soil enzymatic activities was reported due to the increased concentration of vanadium (Xiao et al., 2017). However, in another study, changes in bacterial community structure or soil enzymatic activities were not detected in the paddy soils, in response to the increased concentrations of the heavy metals (Kunito et al., 2017). Chen et al. (2014) showed that long‐term heavy metal pollution decreased the microbial biomass, activity and diversity in paddy soil, particularly in the large‐size fractions. In greenhouse experiment, the viable population of bacteria and fungi was adversely affected by increasing concentration of each heavy metal in soil (Shrivastava et al., 2018). Huaidong, Waichin, Riqing, and Zhihong (2017) showed that long‐term mixed heavy metal pollution affected soil bacterial community structure and reduced alpha diversity in paddy fields. In addition, the abundance and structure of denitrifying microbes in paddy soils was affected by heavy metal pollution but the extent of impact was dependent on soil properties (Liu, Shen, Wu, & Wang, 2018). Thus, there is a need for studies using the high‐throughput sequencing approach for soil microbial community characterization that provides in‐depth analysis of microbial ecology in heavy metal polluted paddy soils.

We hypothesize that a long‐term exposure to the high concentrations of the heavy metals leads to complex changes in the soil microbial community structure, and this process is mostly governed by the replacement of species and to a lesser extent by the differences in community richness. Therefore, the aim of this study was to assess the bacterial community structure and its relationship with the environmental factors in the paddy soils, subject to a long‐term heavy metal stress in the vicinity of nonferrous smelters located in South Korea. In addition, the bacterial community functional changes in response to the heavy metal pollution were assessed using the prediction of the bacterial community functional content from 16S rRNA amplicon data.

## 2 MATERIALS AND METHODS

### 2.1 Study sites and soil sampling

The study was conducted in a nonferrous metal industry area, surrounding the Janghang smelter in Seocheon city, Chungnam, South Korea (Figure S1). The Janghang smelter started its operations in the year 1936, and the smelting furnace was shut down in the year 1989 due to pollution concerns (high content of the heavy metals such as Cd, Cu, Pb, and As). The land, surrounding this smelter, is used for the production of rice. The sampling of the paddy soil was performed in the spring of early 2011. Three composite soil samples, comprising three randomly collected subsamples from a 25 m × 25 m area, were collected from a 0‐ to 30‐cm layer of the three sampling sites, using a spade. Two of these sites were highly contaminated with the heavy metals and were located at distances of 260 m (site S1) and 240 m (site S2) from the smelter, respectively. The third site (site S3) was a paddy field, located at a distance of 3.5 km from the smelter. In total, nine composite samples were collected from the three sampling sites. All the soil samples were kept under cold conditions during transportation to the laboratory. In the laboratory, the samples were homogenized, the plant roots were removed, and finally, the bulk soil was divided into subparts. The subsamples for the microbiological analysis were kept at −20°C, until further analysis. For the chemical analysis, the subsamples were kept at 4°C. The physicochemical characteristics of the soils were measured as described (National Institute of Agricultural Science and Technology [NIAST], 2000). Concentration of As, Cd, Cu, Ni, Pb, and Zn were measured using inductively coupled plasma optical emission spectrometry (ICP‐OES) after acid digestion of 0.5 g of air‐dried soil (NIAST, 2000).

### 2.2 The extraction of soil DNA, PCR, and pyro‐sequencing

Using the Power Soil DNA Isolation Kit (MO BIO Laboratories Inc., Carlsbad, CA, USA), soil genomic DNA was extracted from 0.5 g of soil, following the manufacturer's instructions. The extracted DNA was dissolved in 50 μl of elution buffer and checked for purity and then quantified using Nanodrop 2000 (Thermo Fisher Scientific, Wilmington, DE, USA). The isolated DNA was stored at −20°C for downstream analysis. The extracted genomic DNA was amplified with primers, targeting the V1–V3 hypervariable regions of the bacterial 16S rRNA gene. The samples were PCR amplified and sequenced using a 454 GS‐FLX Titanium Sequencing System by Chunlab (Seoul, South Korea) according to manufacturer instructions. Briefly, the set of primers, containing the Roche 454‐pyro‐sequencing adapter, key, linker, target sequence, and unique barcodes (X) used for the amplification were as follows: 27F 5′‐CCTATCCCCTGTGTGCCTTGGCAGTC‐TCAG‐AC‐GAGTTTGATCMTGGCTCAG‐3′ and 541R 5′‐CCATCTCATCCCTGCGTGTCTCCGAC‐TCAG‐AGAGCTG‐AC‐WTTACCGCGG CTGCTGG–3′. The unique barcodes, identifying the genomic DNA, extracted from each soil sample were 7–11 nucleotides long and attached only to the reverse primers. For each sample, a total volume of 50 μl of the PCR mixture, containing 5 μl of 10× PCR buffer, 1 μl of dNTP (100 mM each), 1 μl of each primer (50 pmol), 1 U of Taq polymerase (Roche, Inc., Basel, Switzerland), 40.8 μl of sterile deionized water, and 1 μl of template DNA, was prepared. The PCR reactions were carried out in a thermocycler (MJ Research, Reno, NV, USA) under the following conditions: initial denaturation at 94°C for 5 min, followed by 25 cycles of denaturation at 94°C for 30 s, annealing at 60°C for 30 s, and elongation at 72°C for 1 min 20 s. Using resin columns, the amplified products were purified and 1 μg of the PCR product from each soil sample was pooled and subjected to pyro‐sequencing, using the standard shotgun sequencing reagents and a 454 GS‐FLX Titanium Sequencing System (Roche, Inc., Basel, Switzerland). Data available from the Dryad Digital Repository: https://doi.org/10.5061/dryad.m35n6.

### 2.3 The analysis of pyro‐sequencing data

The generated sequences were segregated according to their unique barcode sequences. The barcode, the adapter, the key, the linker, and the primer sequences were then trimmed off from the original sequence. Using the Mothur application, the preprocessing and sequence analysis of the FASTA files, containing the trimmed sequences were performed (Schloss et al., 2009). The application included the removal of the redundant sequences, the realignment of the sequences with the compatible database SILVA as a reference, and the filtering for the high‐quality sequences (Pruesse et al., 2007). The candidate chimeric sequences were identified with the UCHIME algorithm inside the Mothur application, using the more abundant sequences from the aligned sequence, itself set as the reference (Edgar, Haas, Clemente, Quince, & Knight, 2011). Following these procedures, the initial number of sequences of 63,991 was reduced to 57,068 for all the samples. The taxonomic assignment of the sequences was made using the Greengenes taxonomic reference set (DeSantis et al., 2006; August 2013 release). The suitable sequences were clustered into operational taxonomic units (OTUs) with CROP at the similarity level of 97% (Hao, Jiang, & Chen, 2011). The final step was performed by random resampling, in order to normalize the sample size to its minimum value (2059 reads).

### 2.4 Statistical analysis

Using the Mothur application, the alpha diversity indices (Shannon and Inverted Simpson's) and the Bray‐Curtis dissimilarity matrix were calculated and constructed. Using the DISTLM program, the distance‐based regression analysis was applied with forward selection procedure and 999 permutations, in order to identify the soil chemical variables that explain the significant amounts of variation in the bacterial community structure (Anderson, 2004) The Principal Coordinate Analysis (PoCA) was calculated in Mothur, using the PCA function and was used to visualize the relationships among the soil samples, based on the bacterial community structure. The differences in the bacterial community structure among the locations were evaluated by applying the one‐way permutational multivariate analysis of variance (PERMANOVA) to the Bray‐Curtis distance matrix (Anderson, Gorley, & Clarke, 2005). Heatmaps were produced using the metagenomeSeq software. Among the three study sites, the differences in the bacterial community structure at the different taxonomic levels were estimated by applying the mvabund package (Wang, Naumann, Wright, & Warton, 2012).

The beta diversity (BD_Total_), calculated as the total variation of the bacterial communities, was obtained by computing the sum‐of‐squares of the relative abundance data for the OTUs and the bacterial genera (Legendre & De Cáceres, 2013). The total sum‐of‐squares of the community composition data was partitioned into the additive components—the species contributions to beta diversity (SCBD) and the local contributions of individual sampling units to beta diversity (LCBD). The variations in the composition of the bacterial communities among the study sites were decomposed into replacement, richness difference, and nestedness indices (Legendre & Gauthier, 2014). The Vikodak software was applied to predict the functional characteristics of the bacterial communities, using the taxonomic abundance, generated from the studied soil samples (Nagpal, Haque, & Mande, 2016). The results obtained from the Vikodak software analysis were further processed with the web‐based software, ClustVis (Metsalu & Vilo, 2015).

## 3 RESULTS

### 3.1 Soil properties

The physicochemical characteristics of the soils, measured using standard laboratory protocols are presented in Table S1 (Tipayno, Kim, & Sa, 2012). The average values of the different heavy metals present in the soils, collected from the three study sites, are presented in Table 1. The observed values of As and Cd for sites S1 and S2 exceeded the threshold and guideline values of 5 mg/kg and 1 mg/kg, respectively (Tóth, Hermann, Szatmári, & Pásztor, 2016), thereby classifying S1 and S2 as the sites polluted with As and Cd. The values of As and Cd were within the limits for site S3, thereby classifying S3 as a normal site. However, the values for the other heavy metals such as Cu, Ni, and Zn for all three sites were lower than the permissible limits.

**Table 1:** Metal and metalloid concentration at the three sampling sites near the smelter

Sites	Metal and metalloid concentration (mg/kg soil)					
	As	Cd	Cu	Ni	Pb	Zn
S1	11.2 ± 1.8^a^	2.3 ± 0.3^ab^	163.98 ± 27.4^a^	2.7 ± 0.1^a^	455.3 ± 135.2^a^	44.6 ± 4.7^a^
S2	16.9 ± 2.9^a^	3.2 ± 0.4^a^	154.8 ± 12.3^a^	2.92 ± 0.5^a^	411.7 ± 50.3^a^	102.6 ± 15.6^a^
S3	1.3 ± 0.07^b^	1.02 ± 0.06^b^	65.33 ± 4.3^b^	3.82 ± 0.4^a^	53.8 ± 1.1^b^	52.6 ± 26.1^a^

Values given are the means of three replicates ±*SE*. Values in each column followed by same letter are not statistically significant at *p* < 0.05.

### 3.2 The bacterial diversity and community structure of the study sites

**Figure 1:**
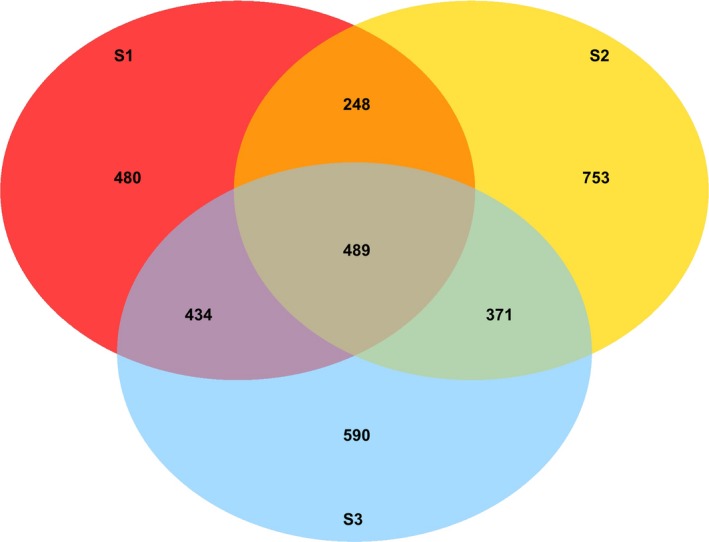
Venn diagram showing the overall overlap of operational taxonomic units (OTU) between the soils collected from three study locations. OTUs are defined at the 97% sequence similarity level

**Figure 2:**
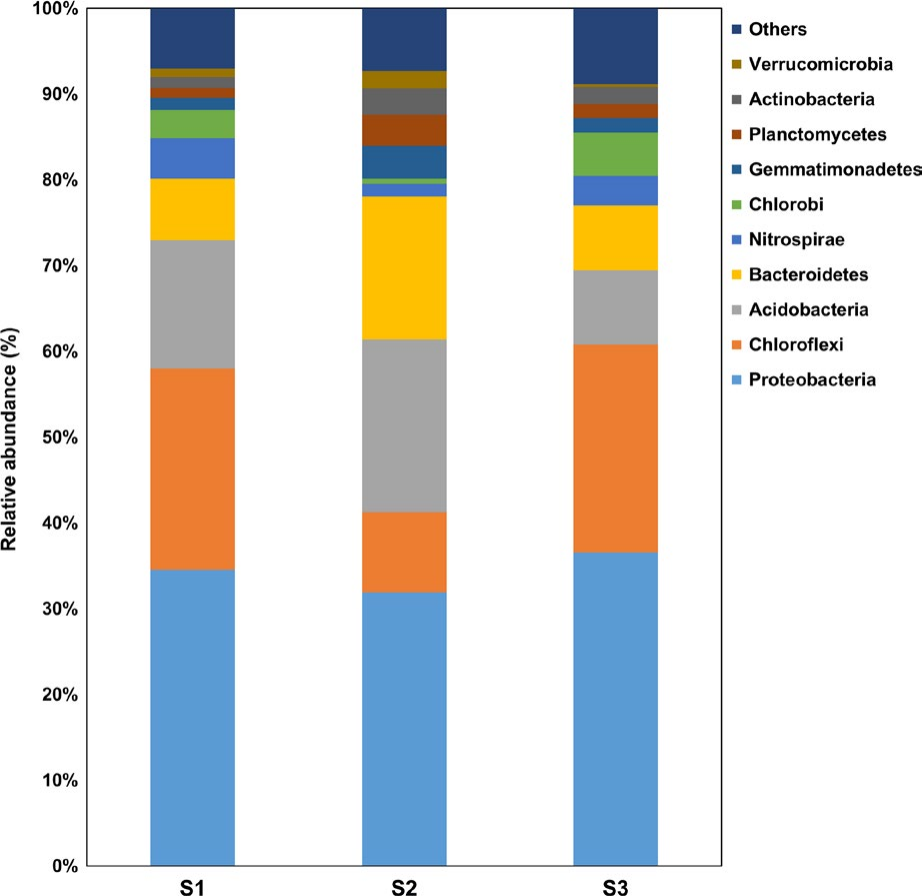
Relative abundance of bacterial phyla at three different studied locations

After sequencing, denoising, normalization, and grouping at the 97% similarity level, a total of 3365 phylotypes (OTUs), comprising all the three study sites were obtained in the dataset. The number of OTUs among the study sites varied significantly (*p *<* *0.05) with the highest variation, observed in site S3 (Table 2). The Shannon and Inverted Simpson's diversity indices did not show any statistically significant differences among the sites. In order to elucidate the differences among the study sites, according to the number of OTUs, a three‐circle Venn diagram was created. The three study sites shared 489 common phylotypes, being 14.5% of the total number of phylotypes (Figure 1). The highest number of unique phylotypes was found at site S2. Across all the study sites, the most abundant bacterial phyla were Proteobacteria (34.3%), Chloroflexi (19.0%), and Acidobacteria (14.6%), followed by the phylum Bacteroidetes (10.5%; Figure 2). The relative abundances of the phyla such as Chlorobi and Chloroflexi were several times lower at S2, the site nearest to the smelter. A total of 248 (7.4% of the total number of phylotypes) unique phylotypes were found at sites S1 and S2, mainly belonging to the phyla such as Proteobacteria (34%), Bacteroidetes (18%), and Acidobacteria (15%). At the genus level, *Flavobacterium*,* Geobacter*,* Pedobacter*,* Koribacter*, and *Opitutus* were found among these phylotypes.

**Table 2:** Summary of bacterial community richness (Sobs) and diversity indices of metal contaminated soils near the smelter

Sites	Sobs	Inverted Simpson index	Shannon index
S1	584 ± 79^c^	142 ± 47^a^	5.6 ± 0.2^a^
S2	757 ± 18^b^	183 ± 31^a^	6.0 ± 0.06^a^
S3	809 ± 4^a^	135 ± 28^a^	6.0 ± 0.04^a^

Values given are the means of three replicates ±*SE*. Values in each column followed by same letter are not statistically significant at *p* < 0.05.

**Figure 3:**
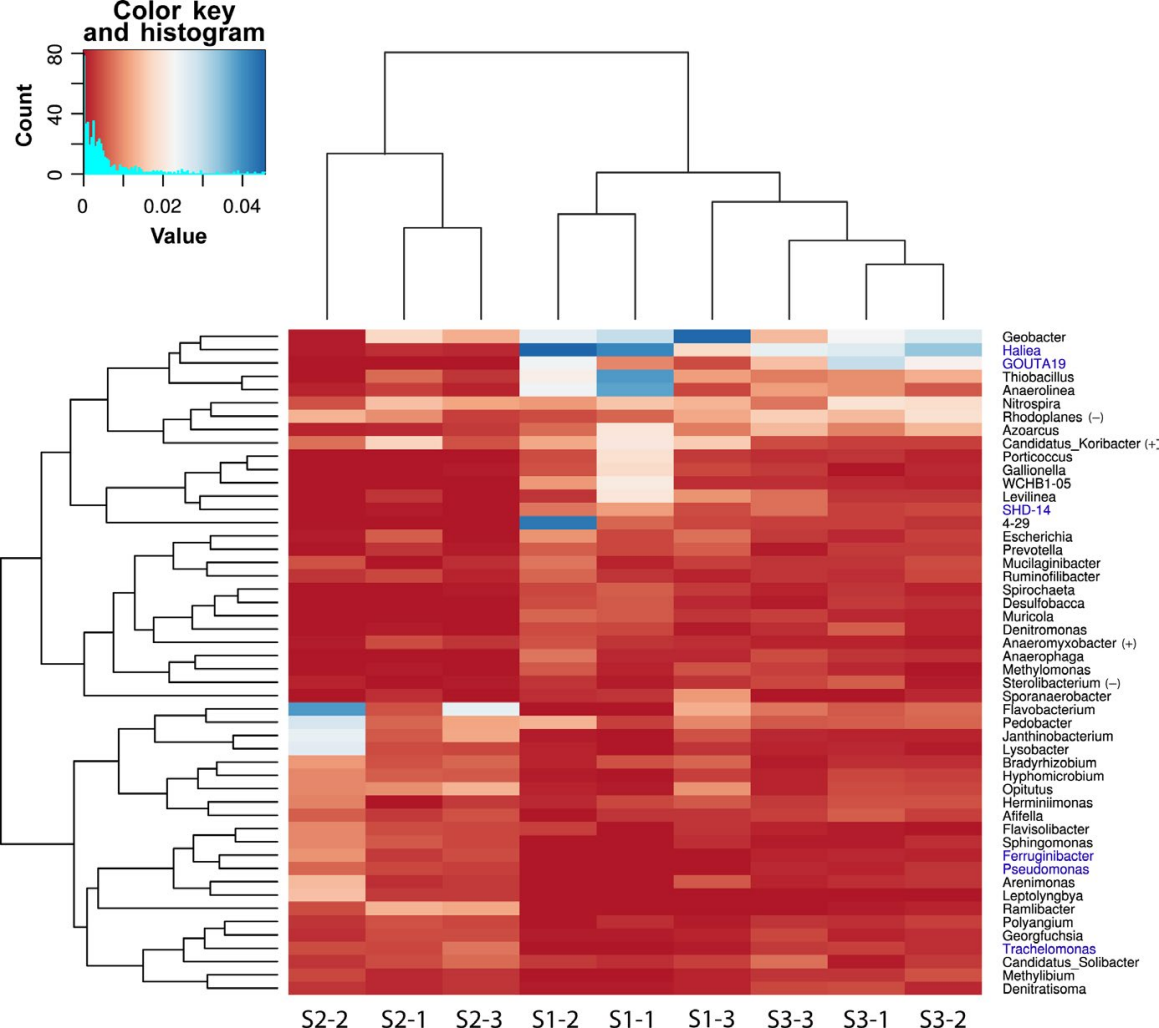
Heatmap showing the clustering of soil samples based on the relative percentage of 16S rRNA gene sequences assigned to 50 most abundant genera. Bacterial genera that were differentially abundant between the study locations according to multivariate ANOVA are shown in blue

Heatmaps, based on the relative abundances of the 50 most abundant bacterial genera revealed that S2, the site nearest to the smelter, was distinct from other two sites S1 and S3 (Figure 3). Among the three study sites, significant differences were observed in the relative abundances of the bacterial genera. According to the results of multivariate ANOVA, the genera such as *Haliea*, GOUTA19, and SHD–14 were abundant at sites S1 and S3, compared to site S2, whereas the genera such as *Pseudomonas*,* Trachelomonas*, and *Ferruginibacter* were relatively more abundant at site S2, compared to sites S1 and S3. The abundances of some of the bacterial genera such as *Geobacter*,* Flavobacterium*,* Anaerolinea*, and *Thiobacillus* varied differently among the study sites.

**Figure 4:**
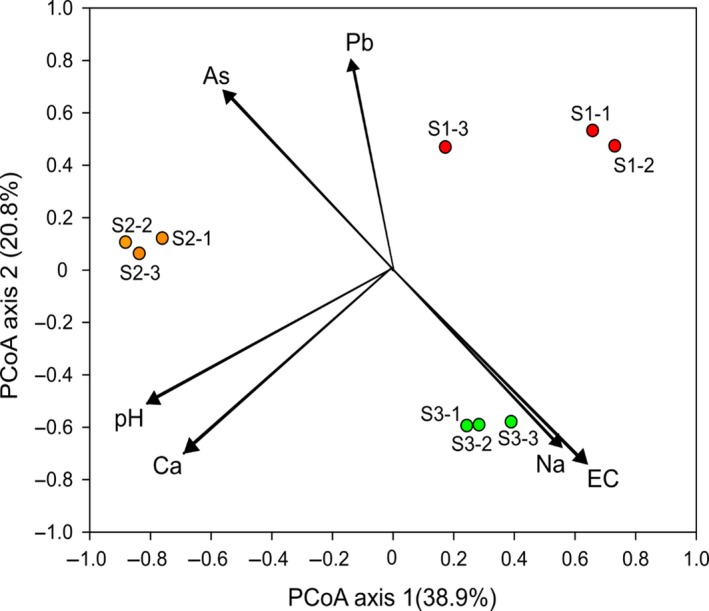
The ordination plot showing a grouping of soil samples according to their bacterial community structure obtained from principal coordination analysis based on the Bray‐Curtis distance matrix. Arrows indicate a correlation of soil chemical variables with first two PCoA axes. Only soil chemical variables are shown that were significantly related to bacterial community variation according to distance‐based regression analysis

The PCA plot, based on the Bray‐Curtis distance matrix, separated the sites clearly and showed the differences among the locations within the study sites (Figure 4). According to the results of PERMANOVA (*p *<* *0.01), the bacterial communities at each of the three sites were distinct from one another. The created Heatmaps, based on the relative abundances of the OTUs, also showed that each site was characterized by a distinct set of bacterial phylotypes (Figure S2).

A statistically significant correlation was observed between the relative abundances of several bacterial phyla and a few soil chemical parameters (Table S2). The soil chemical variables such as pH, electrical conductivity, and the concentrations of sodium and calcium were found to closely correlate with the relative abundances of bacterial phyla.

A similar analysis at the genus level showed that the same soil physicochemical parameters along with phosphorus (P), magnesium (Mg), cation exchange capacity (CEC), zinc (Zn), and aluminum (Al) correlated with the relative abundances of bacterial genera. A few bacterial genera correlated both negatively and positively with the different heavy metals under study (Table S3). The positive and negative correlations obtained were as follows : As correlated positively with *Bacillus* and *Desulfatibacillum* and negatively with *Desulfovirga*; Cd correlated positively with *Bacillus* and negatively with *Desulfatibacillum*,* Desulfoglaeba*, and *Desulfovirga*; Cu correlated positively with *Streptomyces* and negatively with *Desulfatibacillum*,* Desulfococcus*, and *Desulfovirga*; Pb correlated positively with *Desulfatibacillum* and *Desulfovirga* and negatively with *Desulfococcus*, and Ni correlated positively with *Desulfovirga*. The abundances of the bacterial genera such as *Desulfatibacillum* and *Desulfovirga* appeared to have a strong relationship with the heavy metals such as As, Cd, Cu, Pb, and Ni, either positively or negatively.

In the distance‐based regression analysis, the statistically significant soil variables were selected to explain the variations in the bacterial community structure of the soil. The results of this analysis identified the soil parameters such as pH and electrical conductivity to strongly implicate the shift in the community structure with a 56.6% explained variance at *p *<* *0.001 (Table 3). The heavy metals such as As and Pb were seen to be the significant factors, contributing 44.7% in shaping the composition of the bacterial community, while using the soil parameters as the explanatory variables in the stepwise regression model.

**Table 3:** The relationship of soil variables with the composition of bacterial community structure as revealed from the distance‐based regression analysis

Variable type	Variables in model	% variance	*p*‐value
Soil parameters	pH, EC	56.6	<0.001
Cations	Ca, Na	58.3	<0.001
Heavy metals	As, Pb	44.7	<0.01

EC, Electric conductivity; Ca, Calcium; Na, Sodium; As, Arsenic; Pb, Lead.

Looking at the results of the beta diversity partitioning for the three study sites, it appeared that the bacterial community was dominated by replacement, which accounted for 83.8% and 70.3% of the total beta diversity, in the case of the phylotype and the genus level datasets, respectively. The corresponding values for the differences in richness were 16.2% and 29.7%. The LCBD indices were most strongly correlated to the soil As and Pb concentration values (*r* = 0.66 and *r* = 0.77, *p *<* *0.05), in the case of the phylotype dataset. In the case of the genera dataset, the soil As and Cd concentration values correlated to the LCBD indices (*r* = 0.84 and *r* = 0.73, *p *<* *0.05, respectively). Further, the LCBD indices were partitioned into the replacement and richness dissimilarity matrices. The replacement was explained by the soil As and Pb concentration values (26.3% and 53.7%, respectively), while richness was correlated to the soil pH values (29.1% and 31.5% for the phylotypes and the genera, respectively). The SCBD indices for the bacterial genera were statistically significant for the eight genera such as *Ferruginibacter*,* Sphingomonas*,* Arenimonas*,* Flavobacterium*,* Azoarcus*,* Porticoccus*,* Lysobacter*, and *Levilinea*.

### 3.3 The functional potential of the bacterial communities

**Figure 5:**
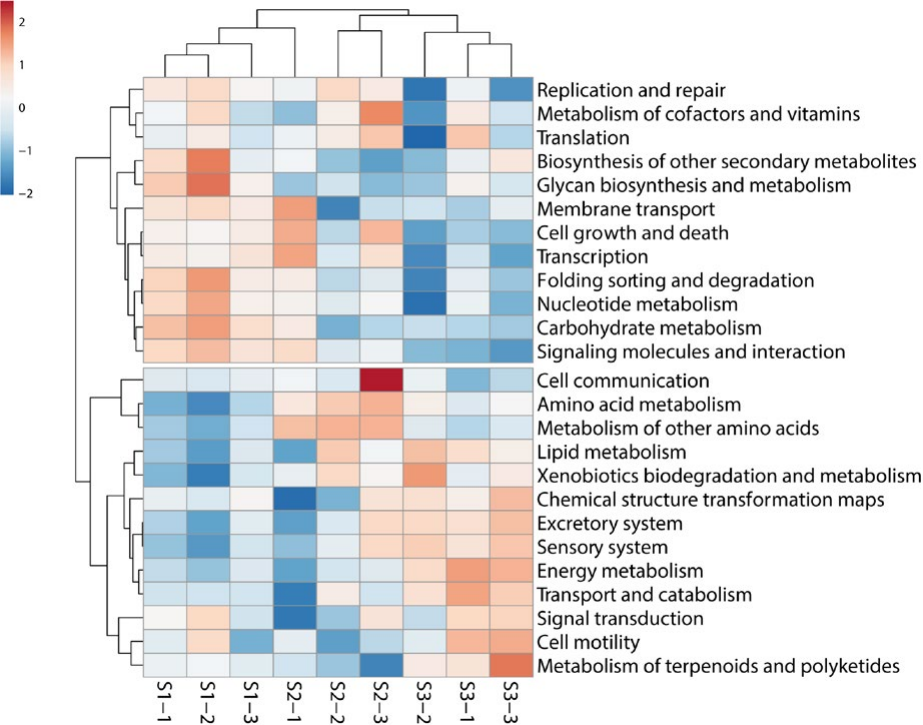
The heatmap showing the clustering of soil samples based on the relative percentage of Vikodak derived bacterial community functional profiles

The results of the clustering of soil samples, based on the “Pathway Abundance Profiles” function in the Vikodak software are shown in Figure 5. The samples from the study site S3 clustered separately from the other two sites S1 and S2. The soil bacterial communities, originating from sites S1 and S2, were characterized by the more abundant enzymes, involved in DNA replication and repair, translation, transcription, and the nucleotide metabolism pathways. At the same time, these sites reduced the relative abundances of the enzymes related to amino acid, lipid, and energy metabolism as well as the biodegradation potential of xenobiotics by the soil bacterial communities. The regression analysis showed that the concentrations of the heavy metals such as As, Cd, and Pb were strongly and positively correlated (*r* = 0.81, *p *<* *0.01) to the relative abundances of the cell growth and death, transcription, and the signaling molecules and interaction pathways (Figure S3). The same metals had strong and negative relationships (*r* = −0.81, *p *<* *0.01) with the pathways, involving transport, catabolism, and the metabolism of terpenoids and polyketides.

## 4 DISCUSSION

In this study, we assessed the response of the bacterial communities to a long‐term heavy metal contamination of the paddy soils along a nonferrous smelter in South Korea. The bacterial communities of the paddy soils under study were characterized by a high relative abundance of the phylum Proteobacteria, followed by the phyla Acidobacteria, Chloroflexi, and that is typical for such type of soils (Ahn et al., 2016). In our study, the phylum Proteobacteria was found to predominate in all the soil samples, regardless of the heavy metal concentration, and the differences in the relative abundances of the bacterial phyla in the study locations can be attributed to the variation in the general soil properties like pH and electrical conductivity. Only in the case of the phylum Chlorobi, a strong negative impact of the soil Cd concentration was revealed. In the forest soils, the abundance of the bacterial phyla such as Chloroflexi, Gemmatimonadetes, and Proteobacteria increased with the increasing level of metal pollution (Azarbad et al., 2015). Previous culture‐dependent and culture‐independent studies of the bacterial communities in the metal contaminated soils, both in the bulk soil and in the rhizosphere, have also shown different results in identifying the dominant bacterial phyla (Berg et al., 2012; Gremion, Chatzinotas, & Harms, 2003). A number of studies have suggested that the soil properties and perhaps, even more, the parent material play larger roles than the metal concentrations in determining the dominant bacterial populations, especially at the phylum level (Ulrich & Becker, 2006).

Although our pyro‐sequencing data are in agreement with our earlier obtained data, using the terminal restriction fragment length polymorphism (T‐RFLP) analysis, the differences in richness and diversity among the study sites are not statistically significant (Tipayno et al., 2012). Previously, other DNA‐based studies have shown the negative or very weak impact of the heavy metals on the microbial diversity in the soils (Bamborough & Cummings, 2009; Gans, Wolinsky, & Dunbar, 2005; Sheik et al., 2012). In some cases, an increase in the bacterial alpha diversity indices in the heavy metal polluted soils has also been observed (Hong et al., 2015). The diversity of bacterial community in the metal polluted soils estimated using DNA‐based methods may not change due to fact that some bacterial community members might display low activity profiles due to pollution while RNA‐based methods may reveal reduction in community diversity due heavy metals (Nunes et al., 2016).

Previous studies have shown that the adaptation of the bacterial communities to the heavy metals in the soil may occur through physiological adaptation, the replacement of the sensitive species by the more tolerant ones, and adaptive mutations (Azarbad et al., 2015). Using the variance partitioning approach, we identified the replacement of species as the main process behind the observed differences among the studied bacterial communities. Moreover, the replacement process was explained by the concentration of the heavy metals such as As and Pb, while the differences in richness of the bacterial communities were linked to the variations in the soil pH. The above findings imply that in the case of the metal contaminated paddy soils, the bacterial species, more sensitive to the heavy metals and lacking metal resistance mechanisms, are replaced by the more tolerant species. The applied data analysis approach also enabled the identification of bacterial taxa that contributed to the observed differences among the studied bacterial communities most significantly and included the metal‐tolerant genera such as *Arenimonas*,* Ferruginibacter*,* Flavobacterium*, and *Lysobacter* (Chen, Shi, & Wang, 2012; Puopolo et al., 2016; Shah, Collins, Walker, & Shah, 2014). In addition, the data analysis identified that the bacterial genus *Bacillus* was positively related to the increased levels of the heavy metals in the soil. In fact, the As‐resistant *Bacillus* strains have been shown to reduce As(V) to As(III) (Ruta et al., 2011). In order to delineate the importance of different evolutionary processes (lateral gene transfer, selective survival of heavy metal resistant populations, accumulation adaptive mutations) in mediating the response of a soil microbial community to heavy metal contamination, amplicon‐based sequencing should be combined with single‐cell, gene, and genome‐centric metagenomic sequencing (Hemme et al., 2016; Starnawski et al., 2017).

Sulfate reduction is an important process in the attenuation and remediation of the acid mine drainage (AMD) impacted as well as the radionuclide and metal contaminated environments. Dissimilatory sulfate‐reducing bacteria have been shown to transform the metal ions into insoluble and chemically inert forms, producing carbonates and sulfides, which further precipitate the metal cations or reduce metal oxycations and oxyanions, thereby stabilizing these toxic metal ions as solid metal sulfides (Sitte et al., 2010). Our results indicate that the increase in the soil metal pollution is accompanied by the reduction in the abundance of the sulfate‐reducing bacterial genera that negatively affects the immobilization of the heavy metals in the polluted soils. In addition to the sulfate‐reducing bacteria, the abundance of the bacterial genera such as *Rhodoplanes* was affected by the heavy metals in the soil. Some members of the genus *Rhodoplanes* have been classified as purple nonsulfur bacteria and have been considered important to the soil functioning as they can fix nitrogen and produce indole‐3‐acetic acid (IAA) and 5‐aminolevulinic acid (ALA; Sun, Xiao, Ning, Xiao, & Sun, 2015). Therefore, the decrease in the relative abundance of the genus *Rhodoplanes* may have a negative impact on the soil fertility.

A long‐term exposure to the heavy metals not only changes the soil bacterial community structure but may also affect the functional properties of the communities. For characterizing the phylogenetic distribution of the bacterial communities, 16S rRNA gene‐based analysis is a powerful tool that can be complemented with the prediction of the functional capabilities of a microbial community, based on this marker gene data. Using the prediction of the bacterial community functions, we found that the soil bacterial communities at the heavy metal polluted locations were characterized by the more abundant enzymes, involved in DNA replication and repair, translation, transcription, and the nucleotide metabolism pathways. Similar changes in the bacterial community functions due to the heavy metal contamination have been recorded in other studies. A higher expression of the pathways (transposition or the transfer of genetic material and membrane transport), potentially involved in the metal resistance mechanisms, has also been found in the heavy metal contaminated soils of an abandoned Pb‐Zn mine (Epelde et al., 2015).

At the same time, in our study, the heavy metals reduced the relative abundances of the enzymes related to amino acid and lipid metabolism as well as the biodegradation potential of xenobiotics by the soil bacterial communities. This finding is in concordance with the study of the heavy metal contaminated bacterial communities in groundwater that showed the reduced capacity for degrading complex carbohydrates and the mineralization of xenobiotics under pollution stress (Hemme et al., 2015; Singh et al., 2014). One of the adaptation mechanisms of the bacterial communities to the heavy metals in the soil is a stress response. The elevated DNA replication and repair, as well as the signaling molecules and the interaction pathways in the bacterial communities at the polluted sites, could be related to the formation of the hazardous reactive oxygen species (ROS) and cellular signaling pathways, that both are important in the bacterial resistance mechanisms toward the heavy metals (Prabhakaran, Ashraf, & Aqma, 2016).

We found that despite the differences in the taxonomic composition of the bacterial communities between the two metal polluted locations, the functional potential of these communities appears to be similar. While considering these results, we have to take into account that the prediction accuracy of the community functions depends on the level of correspondence between the organisms in the16S rRNA gene and the genome databases.

The present study is based only on the 16S rRNA gene amplicon data, which are generally more robust for the analysis of community ecology and provide more accurate estimates of biodiversity at the different taxonomic scales, compared to the shotgun metagenomic approaches (Tessler et al., 2017). However, up to 10% of the microbial sequences in the environment might be missed from the classical PCR‐based 16S rRNA gene surveys (Eloe‐Fadrosh, Ivanova, Woyke, & Kyrpides, 2016). Another limitation of our study is that our results are based on the assessment of the bacterial communities in the long‐term metal polluted soils and the way these communities contrast to the unpolluted soils. This means that the initial status and the bacterial community structure of the polluted soils are assumed to be similar to the soils, currently used as the reference locations. In order to get deeper insights into the alterations of the diversity of the soil bacterial communities as well as the metabolic functions due to the heavy metal pollution, the amplicon and shotgun metagenomic approaches should be combined in the future studies.

## 5 CONCLUSION

Our results indicate that the soil bacterial communities had adapted to the elevated concentrations of heavy metals in the polluted paddy soils, as evidenced by the changes in the relative abundances of the particular groups of microorganisms at the different levels of taxonomic resolution. Also, the functional potential of the soil bacterial communities was altered due to the heavy metal contamination. The dissimilarities in the soil bacterial community assembly between the heavy metal contaminated sites were due to site‐specific differences in soil chemical properties but community functional profile was mainly determined by heavy metal concentration values in the soil. The adaptation of the bacterial communities to the heavy metal contamination was predominantly attributed to the replacement process, while the changes in community richness were linked to the variations in the soil pH values.

## CONFLICT OF INTEREST

None declared.

## AUTHOR CONTRIBUTIONS

SCT, SS, JT, MT, and TS performed conceptualization. SCT, SS, JT, PC, ME, KO, YK, and KK performed data acquisition. SCT, SS, PC, YK, KK, JKP, ME, KO, MT, and TS performed data analysis. TS, JT, and MT performed supervision. SCT, SS, JT, JKP, MT, ME, KO, and TS performed article draft. SCT, SS, PC, YK, KK, and TS performed review and editing.
