# Supplementary material for: The bacterial community structure and functional profile in the heavy metal contaminated paddy soils, surrounding a nonferrous smelter in South Korea
Source: Ecol Evol. 2018 May 20;8(12):6157–68. doi: 10.1002/ece3.4170 (PMC6024150; doi:10.1002/ece3.4170)
Supplement: Supplementary file 1 [file ECE3-8-6157-s001.docx]

**Supporting Information**

**Fig S1.** Sampling locations of heavy metal and metalloid contaminated sites in the vicinity of Janghang smelter.

**Fig S2.** The heatmap showing the clustering of soil samples based on the relative percentage of 16S rRNA gene sequences assigned to 50 most OTUs are defined at 97% sequence similarity level.

**Fig S3.** The heatmap showing the correlation of bacterial community functional profiles with the soil heavy metal concentration. Abbreviation: Metsum – sum of the heavy metals.

**Table S1.** Chemical properties of soils from the three sampling locations along the Janghang smelter. Abbreviations: EC – Electric conductivity, T-N – Total Nitrogen, CEC- Cation Exchange Capacity.Least significant difference (LSD) was used for the mean separation at 0.05.

**Table S2.** Correlation between bacterial phyla and soil chemical properties of the sampled sites. **p*< 0.05, ***p*<0.01

**Table S3.** Correlation between bacterial genera and soil chemical properties of the sampled sites. **p*< 0.05, ***p*<0.01

**Fig S1.**

**
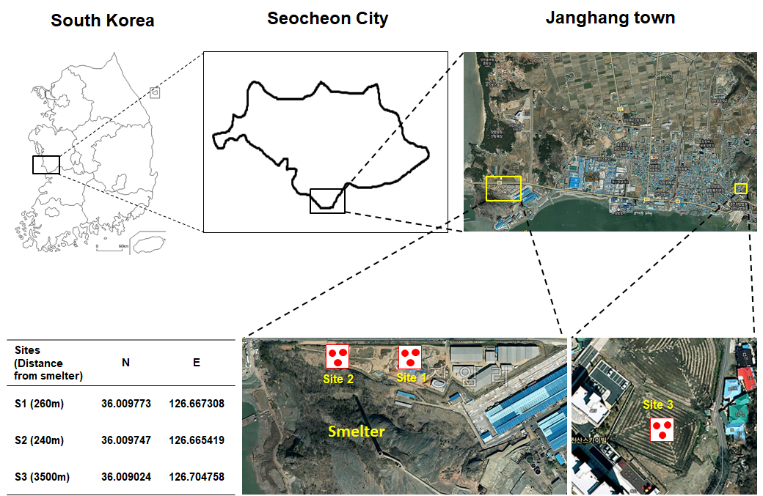
**

**Fig S2.**

**
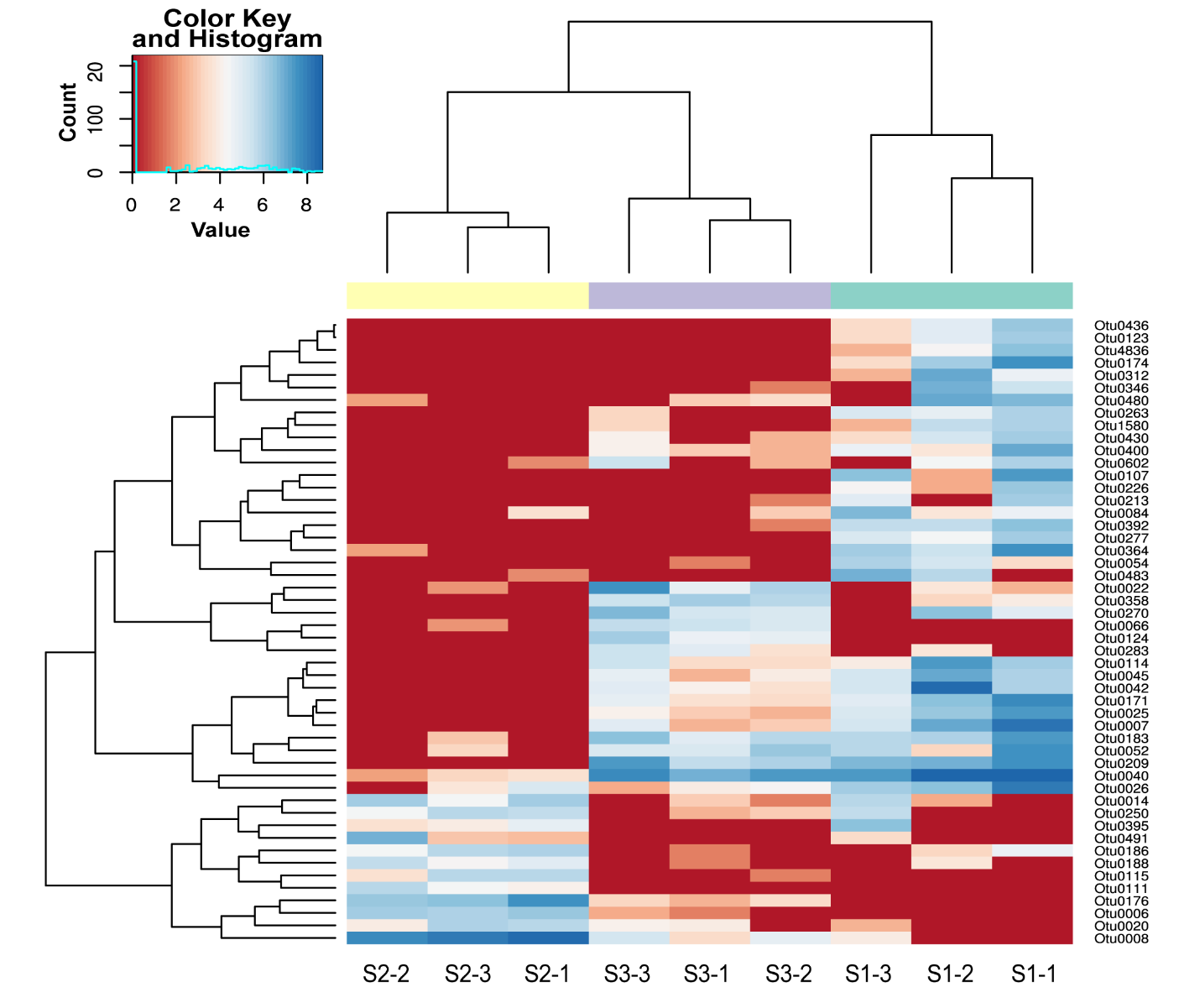
**

**Fig S3.**

**
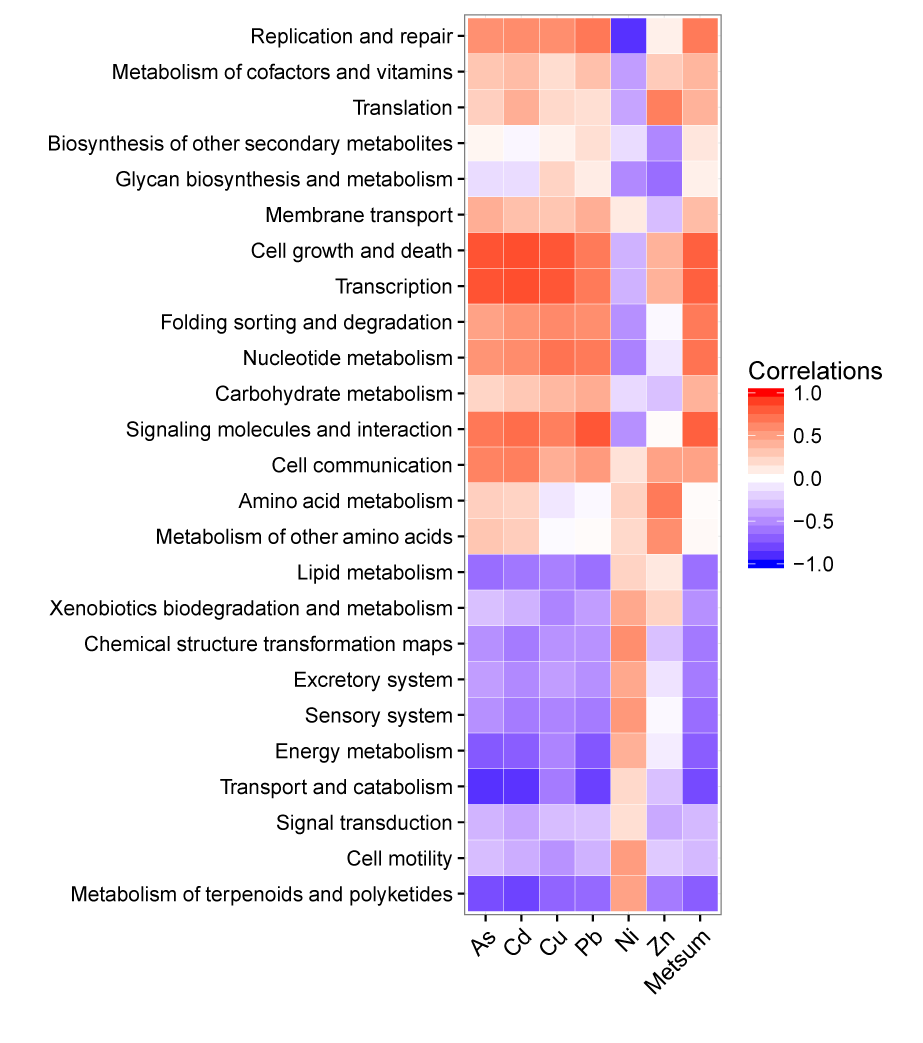
**

**Table S1**

| Sites | EC (dS/m) | pH  (1:5) | T-N  % | P_2_O_5_ (mg kg^-1^) | Exchangeable cations | | | | | CEC  (cmol kg-^1^) |
| --- | --- | --- | --- | --- | --- | --- | --- | --- | --- | --- |
|  |  |  |  |  | **K** | **Ca** | **Mg** | **Na** | **Al** |  |
|  |  |  |  |  | **cmol kg^-1^** | | | | |  |
| S1 | 0.25 | 5.94 | 0.13 | 1085.5 | 0.38 | 5.8 | 4.32 | 0.37 | 0.02 | 14.41 |
| S2 | 0.11 | 7.2 | 0.16 | 3722.1 | 0.52 | 10.6 | 2.97 | 0.19 | 0.12 | 16.81 |
| S3 | 0.53 | 6.8 | 0.26 | 2843.9 | 0.56 | 10.0 | 3.29 | 0.95 | 0.03 | 17.12 |
| LSD | 0.20 | 0.88 | 0.05 | 1896.6 | 0.14 | 1.82 | 0.67 | 0.36 | 0.27 | 2.05 |

**Table S2**

| **Phylum** | **pH** | **EC** | **Na** | **Cd** | **Ca** | **Al** | **Zn** |
| --- | --- | --- | --- | --- | --- | --- | --- |
| ***Chloroflexi*** | - | - | 0.70* | - | - | - | - |
| ***Acidobacteria*** | - | -0.82** | -0.71* | - | - | - | - |
| ***Bacteroidetes*** | 0.71* | -0.79* | -0.71* | - | - | - | - |
| ***Chlorobi*** | - | 0.72* | 0.85** | -0.67* | - | - | - |
| ***Gemmatimonadetes*** | 0.86** | -0.70* | - | - | 0.71* | 0.69* |  |
| ***Planctomycetes*** | 0.82** | - | - | - | - | - | 0.82** |
| ***Planctomycetes*** | - | - | - | - | - | - | - |
| ***Actinobacteria*** | 0.78* | -0.67* | -0.70* | - | - | - | - |
| ***Verrucomicrobia*** | - | -0.72* | - | - | - | - | - |

**Table S3**

| **Genus** | **pH** | **T-N** | **EC** | **P** | **Na** | **Ca** | **Mg** | **CEC** | **Zn** | **As** | **Cd** | **Cu** | **Al** | **Ni** | **Pb** | **K** |
| --- | --- | --- | --- | --- | --- | --- | --- | --- | --- | --- | --- | --- | --- | --- | --- | --- |
| ***Alistipes*** | - | - | - | - | 0.70^*^ | - | - | - | - | - | - | - | - | - | - | - |
| ***Alkalitalea*** | -0.80^**^ | - | 0.69^*^ | - | - | -0.70^*^ | - | - | - | - | - | - | - | - | - | - |
| ***Anaerolinea*** | - | - | 0.85^**^ | - | 0.86^**^ | - | - | - | - | - | - | - | - | - | - | - |
| ***Anaerophaga*** | - | - | 0.73^*^ | - | 0.86^**^ | - | - | - | - | - | - | - | - | - | - | - |
| ***Angiococcus*** | - | - | - | - | - | - | -0.69^*^ | - | - | - | - | - | - | - | - | - |
| ***Arthrobacter*** | - | - | - | - | - | - | - | 0.77^*^ | 0.75^*^ | - | - | - | - | - | - | - |
| ***Azoarcus*** | - | - | 0.84^**^ | - | 0.73^*^ | - | - | - | - | - | - | - | - | - | - | - |
| ***Bacillus*** | - | - | - | - | -0.81^**^ | - | - | - | 0.68^*^ | 0.70^*^ | 0.74^*^ | - | - | - | - | - |
| ***Cystobacter*** | 0.84^**^ | - | - | 0.89^**^ | - | 0.91^**^ | -0.81^**^ | 0.71^*^ | 0.84^**^ | - | - | - | 0.68^*^ | - | - | - |
| ***Denitromonas*** | - | - | - | - | 0.79^*^ | - | - | - | - | - | - | - | - | - | - | - |
| ***Desulfatibacillum*** | - | - | 0.77^*^ | - | 0.90^**^ | - | - | - | - | -0.87^**^ | -0.89^**^ | -0.74^*^ | - | - | -0.84^**^ | - |
| ***Desulfococcus*** | - | - | - | - | 0.78^*^ | - | - | - | - | - | - | -0.82^**^ | - | - | -0.70^*^ | - |
| ***Desulfoglaeba*** | - | - | 0.93^**^ | - | 0.85^**^ | - | - | - | - | - | -0.68^*^ | -0.67^*^ | - | - | - | - |
| ***Desulfovirga*** | - | - | - | - | - | - | - | - | - | -0.80^*^ | -0.79^*^ | - | - | 0.73^*^ | -0.86^**^ | - |
| ***Dongia*** | 0.81^**^ | - | - | 0.68^*^ | - | 0.77^*^ | -0.80^*^ | - | 0.74^*^ | - | - | - | 0.80^*^ | - | - | - |
| ***Dyella*** | - | - | - | - | - | - | 0.76^*^ | - | - | - | - | - | - | - | - | - |
| ***Escherichia*** | - | - | - | - | - | - | -0.72^*^ | -0.72^*^ | - | - | - | - | - | - | - | -0.67^*^ |
| ***Ferruginibacter*** | 0.93^**^ | - | - | 0.83^**^ | - | 0.83^**^ | -0.71^*^ | 0.77^*^ | 0.75^*^ | - | - | - | - | - | - | - |
| ***Flavobacterium*** | 0.76^*^ | - | - | - | -0.68^*^ | - | - | - | - | - | - | - | - | - | - | - |
| ***Georgfuchsia*** | 0.86^**^ | - | - | 0.68^*^ | - | - | - | -0.95^**^ | - | - | - | - | 0.93^**^ | - | - | - |
| ***Haliea*** | -0.82^*^ |  | - | - | - | - | - | - | - | - | - | - | - | - | - | - |
| ***Herbaspirillum*** | - | 0.68^*^ | - | 0.68^*^ | - | 0.72^*^ | - | 0.74^*^ | - | - | - | - | - | - | - | 0.88^**^ |
| ***Herminiimonas*** | -0.79^*^ | - | - | - | - | - | 0.76^*^ | - | - | - | - | - | - | - | - | - |
| ***Holophaga*** | - | - | 0.71^*^ | - | - | - | - | - | - | - | - | - | - | - | - | - |
| ***Levilinea*** | -0.75^*^ | - | - | - | - | - | - | -0.73^*^ | -0.70^*^ | - | - | - | - | - | - | - |
| ***Lysobacter*** | 0.78^*^ | - | -0.82^**^ | - | -0.69^*^ | - | - | - | - | - | - | - | - | - | - | - |
| ***Marinilabilia*** | -0.74^*^ | - | - | - | 0.73^*^ | - | 0.74^*^ | - | - | - | - | - | -0.70^*^ | - | - | - |
| ***Marmoricola*** | - | - | - | - | - | 0.73^*^ | - | 0.80^*^ | - | - | - | - | - | - | - | - |
| ***Methylomicrobium*** | - | - | - | -0.67^*^ | - | -0.77^*^ | - | -0.77^*^ | -0.72^*^ | - | - | - | - | - | - | - |
| ***Muricola*** | -0.88^**^ | - | - | -0.71^*^ | - | -0.80^*^ | 0.73^*^ | - | -0.78^*^ | - | - | - | -0.68^*^ | - | - | - |
| ***Nitrobacteria*** | 0.77^*^ | - | - | 0.70^*^ | - | 0.87^**^ | - | 0.85^**^ | 0.75^*^ | - | - | - | - | - | - | - |
| ***Owenweeksia*** | - | - | - | - | - | - | 0.72^*^ | - | - | - | - | - | - | - | - | - |
| ***Polyangium*** | 0.74^*^ | - | - | 0.71^*^ | - | - | -0.98^**^ | - | - | - | - | - | 0.88^**^ | - | - | - |
| ***Porticoccus*** | -0.92^**^ | - | - | -0.71^*^ | - | -0.71^*^ | 0.83^**^ | - | - | - | - | - | -0.74^*^ | - | - | - |
| ***Prevotella*** | -0.78^*^ | - | - | -0.68^*^ | - | - | 0.76^*^ | - | - | - | - | - | - | - | - | -0.70^*^ |
| ***Pseudoburkholderia*** | 0.82^**^ | - | -0.83^**^ | 0.67^*^ | -0.74^*^ | 0.69^*^ | - | - | 0.73^*^ | - | - | - | - | - | - | - |
| ***Pseudomonas*** | 0.85^**^ | - | - | 0.73^*^ | - | - | -0.80^*^ | - | - | - | - | - | - | - | - | - |
| ***Roseomonas*** | 0.72^*^ | - | -0.67^*^ | - | -0.81^**^ | - | - | - | - | - | - | - | 0.68^*^ | - | - | - |
| ***Rubrivivax*** | 0.67^*^ | - | - | - | - | - | - | - | 0.83^**^ | - | - | - | - | - | - | - |
| ***Sphingomonas*** | 0.78* | - | - | - | -0.70* | - | - | - | - | - | - | - | - | - | - | - |
| ***Spirochaeta*** | -0.89^**^ | - | - | -0.72^*^ | - | -0.67^*^ | 0.82^**^ | - | - | - | - | - | -0.71^*^ | - | - | - |
| ***Streptomyces*** | - | -0.74^*^ | - | - | - | - | - | - | - | - | - | 0.73^*^ | - | - | - | - |
| ***Trachelomonas*** | 0.98^**^ | - | - | 0.81^**^ | - | 0.86^**^ | -0.75^*^ | 0.68^*^ | 0.73^*^ | - | - | - | - | - | - | - |
